# Supplementary material for: Notch1 Phase Separation Coupled Percolation facilitates target gene expression and enhancer looping
Source: Sci Rep. 2024 Sep 19;14:21912. doi: 10.1038/s41598-024-71634-6 (PMC11413390; doi:10.1038/s41598-024-71634-6)
Supplement: Supplementary file 43 — Supplementary Information 35. [file 41598_2024_71634_MOESM43_ESM.pdf]

Extendend Data and Supplemental Information Captions

Extended Data Figure 1: Molecular Dynamic Simulation Of The Notch1 Intracellular Domain (AA1754-AA2555)

A) Representative heatmap of the root means squared deviation(RMSD), in angstroms(Å), of individual residues following molecular dynamic simulation in GROMACS2 with RMSD calculated in VMD over 2000 frames spread over the simulation. B) Domain RMSD plots over time looking at the structural movement of either the RAM and Ankyrin repeat domain(1754-2119, blue), the TAD domain and c-terminal tail(2120-2555, green), or the entirety of the Notch1 intracellular domain(1754-2555, purple) C) N1ICD structural analysis using Prediction of prion-like domains (PLD), Net charge per residue (NCPR), Fraction of charged residues (FCR), and hydrophobicity analysis.

Extended Data Figure 2: Effect of 1,6-Hexanediol treatment on Notch1 Condensates

A) Formation of hollow cavities(arrows) in *in-vitro* droplets at 250mM NaCl. Brightfield(top), green fluorescence (bottom). Scale Bar 10µm. B) Isolated NICD::GFP treated either with control (left) or 1,6-Hexanediol treatment, concentration used was 100um NICD::GFP in 150mM NaCl. Scale Bar 50µm C) Live imaging of N1ICD in OptoNotch activated HEK293 cells before and six seconds after treatment with either vehicle(DMSO) or 1,6-Hexandiol. Scale bar 10µm. D) Quantification of the number of N1ICD foci per nucleus in cells from panel C, showing that 1,6-Hexandiol treatment results in a near-complete loss of intranuclear N1ICD foci from 29.7(+/-30.1) in vehicle control cells to 0.6(+/-0.8) in 1,6-Hexanediol-treated cells. N=1000 cells per condition. E) Quantification of the total intensity of Notch1 in OptoNotch-activated HEK293 cells from panel C, showing that treatment with 1,6-Hexanediol does not significantly impact total Notch1 intensity in comparison to vehicle control treated cells, 2643(+/-877) and 2566(+/-599) A.U of Notch1, respectively. N= 1000 cells per condition. $<0.1 student t-test. See Source Data Extended Figure 2.

Extended Data Figure 3: Antibody validation and comparison of commercial Notch1 antibodies

A) Notch1 immunostainings from HEK293 cells either plated on control(top), or DeltaMAX(bottom) using CST antibody. B) Total nuclear Notch1 protein from panel A showing control and DeltaMAX treated cells respectively had 946(+/-312) and 1276(+/-462) A.U of Notch1. N= 5000 nuclei per condition. C) Volume of individual nuclear foci from panel A showing control and DeltaMAX treated cells respectively form Notch1 foci that are on average 0.141(+/-0.153), and 0.554(+/-0.335). N= 51000 foci per condition. D) Total Individual Fluorescence per foci from panel A showing control and DeltaMAX treated cells respectively have foci that have 37(+/-38.7) and 65.6(+/-40.1) A.U of Notch1. N= 51000 foci per condition. E) Notch1 immunostainings from HEK293 cells either plated on control (Top) or DeltaMAX (Bottom) using DSHB antibody. F) Total Nuclear Notch1 protein per Cell from panel E showing control and DeltaMAX treated cells respectively had 1891.5(+/-581.1) and 3194.3(+/-3343.9) A.U of Notch1. N= 3500 nuclei per condition. G) Volume of individual nuclear foci from panel E showing control and DeltaMAX treated cells respectively form Notch1 foci that are 0.142 $µm^3$ (+/-0.238) and 0.235 $µm^3$ (+/-0.635) average. N= 4000 foci per condition. H) Total Individual Fluorescence per foci from panel E showing control and DeltaMAX treated cells respectively have foci that have 69.1(+/-109.7) and 99.3(+/-249.88) A.U of Notch1. N= 4000 foci per condition. I) Endogenous Notch1 fluorescence immunostainings in T-ALL cells treated either

with DMSO or GSI using CST antibody. J) Total amount of Nuclear Notch1 protein per cell from panel I showing DMSO and GSI treated cells have 1159(+/-507) and 774(+/-460) A.U of Notch1, respectively. N= 31000 cells measured per condition. K) Volume of individual nuclear foci from panel I showing DMSO and GSI treated cells form Notch1 foci that are on average 0.658μm^3(+/-0.636) and 0.236μm^3(+/-0.127) respectively. N= 54000 foci measured per condition. L) Total Fluorescence per individual nuclear foci from panel I showing DMSO and GSI treated cells have foci of 57.9(+/-65.3) and 25.6 (+/-14.2) A.U of fluorescence intensity for Notch1. N= 54000 foci measured per condition. M) Endogenous Notch1 fluorescence immunostainings in T-ALL cells treated either with DMSO or GSI using DSHB antibody. N) Total amount of Nuclear Notch1 protein per cell from panel M showing DMSO and GSI treated cells have 3516(+/-1571) and 1556(+/-680) A.U of Notch1, respectively. N= 4000 cells measured per condition. O) Volume of individual nuclear foci from panel M showing DMSO and GSI treated cells form Notch1 foci that are on average 0.564μm^3(+/-0.502) and 0.235μm^3(+/-0.128) respectively. N= 12000 foci measured per condition. P) Total Fluorescence per individual nuclear foci from panel M showing DMSO and GSI treated cells have foci of 40.5(+/-43.7) and 21.1(+/-11.6) A.U of fluorescence intensity for Notch1. N= 12000 foci measured per condition. *p<0.01 One-way ANOVA+Tukey post-hoc, $p<0.01 student t-test. See Source Data Extended Figure 3

Extended Data Figure 4: Cleavage of Notch1 in the absence and presence of exogenous, surface-immobilized DeltaMAX ligand:

A) Western blot of HEK293 cells following nuclear/cytoplasmic fractionation in the absence or presence of GSI inhibitor. Activated Notch1 antibody staining shows cleavage of endogenous Notch1(left two lanes), with cleaved Notch1 present at high levels in the nucleus compared to the cytoplasm. GSI treatment inhibits Notch1 cleavage, as shown by the loss of the activated Notch1 band(Right two lanes). Lamin A/C and β–tubulin was used as loading controls for the nuclear and cytosolic fractions, respectively. B) Western blot of HEK293 cells cultured either in the absence of exogenous ligand (control) or with immobilized DeltaMAX ligand. Activated Notch1 antibody staining shows a band corresponding to cleaved endogenous Notch1, which is present at high levels in the nucleus compared to the cytoplasm (control, lanes one and three). Treatment with surface-immobilized DeltaMAX ligand activates Notch1 cleavage over baseline control, as shown by the increase in the abundance of activated Notch1 in the nuclear fraction(DeltaMAX, lanes two and four). Lamin A/C and β–tubulin was used as loading controls for the nuclear and cytosolic fractions, respectively. N=3 for each blot.

Extended Data Figure 5: Comparison of OptoNotch condensate properties in untreated and DeltaMAX-activated cells.

A) Immunostaining against Notch1 in T-ALL cells, DeltaMAX-plated HEK293 cells, and GSI-treated OptoNotch activated HEK293 cells using a secondary 647 that has been re-colourized to green. Scale bar 10μm. B) Quantification of Hes1 expression by qPCR in either GSI treated, DeltaMAX plated, or OptoNotch activated HEK293 cells, showing a -0.736(+/-0.839), 13.777(+/-0.850), and 6.465(+/-1.385) fold change in expression, respectively. C) Quantification of Total Nuclear Notch1 showing T-ALL cells, DeltaMAX plated HEK293 cells, or OptoNotch activated HEK293 cells have 1782(+/-2137),1463(+/-1005) and 3841(+/-3181) A.U of Notch1, respectively. N=3000 cells per condition D) Quantification of the total volume per foci from T-ALL cells, DeltaMAX plated HEK293 cells, or OptoNotch activated HEK293 cells form Notch1 foci that are on average 0.640 μm^3 (+/-0.506), 0.551 μm^3 (+/-0.343), and 0.554 μm^3 (+/-0.406), respectively. N= 5000 foci per condition. E) Quantification of the total Intensity of Individual

Notch1 foci from T-ALL cells, DeltaMAX plated HEK293 cells, or OptoNotch activated HEK293 cells form Notch1 foci that have an average fluorescence intensity of 26.1(+/-49.8), 18.3(+/-37.1), 23.3(+/-56.4) A.U., respectively. N= 5000 foci per condition. F) Quantification of the total number of Notch1 foci per cell showing T-ALL cells, DeltaMAX plated HEK293 cells, or OptoNotch activated HEK293 cells form 8.23(+/-4.6), 17(+/-21.4), and 32.2(+/-29.3) Notch1 Foci per cell, respectively. N= 3000 cells per condition. *p<0.01 One-way ANOVA+Tukey post-hoc. See Source Data Extended Figure 5.

Extended Data Figure 6: Super-Resolution Quantitative Image Rating And Reporting Of Error Locations(SQUIRREL) Analysis

A) Original representative confocal image acquired using spinning disc confocal microscopy (Top left). Post-SRRF analysis of confocal images (Top right). Convolved SRRF image for error mapping of confocal image (Bottom left). Error map of SRRF data showing a Resolution Scaled Pearson's(RSP) of 0.974 and an average resolution scaled error (RSE) of 3.732 (Bottom right). Scale bar 5 µm. B) Dot plot of average RSE value output by individual SRRF images. N=250. C) Dot plot of RSP value output by individual SRRF images. N=250 See Source Data Extended Figure 6.

Extended Data Figure 7: Additional T-ALL N1ICD Molecular Condensates SRRF Imaging .

T-ALL cells immunostained against Notch1 Imaged using SRRF microscopy showing the formation of ring-like structured N1ICD condensates. Scale Bar 500nm

Extended Data Figure 8: Addition SRRF Analysis of Notch1 Nuclear Condensates

A) Direct size comparison between an initial focus (pre-bleaching, pink) to its final state following photo-bleaching recovery from Figure 3.F. B) Direct size comparison between an initial focus (pre-bleaching, pink) to its final state following photo-bleaching recovery from Figure 3.G. C) SRRF images of an intranuclear N1ICD focus showing a decrease in size over time. D) SRRF images of an intranuclear N1ICD focus showing an increase in size over time. E) Overlay of the initial (0 minutes; pink), and final image (7 minutes; green) from Panel C. F) Overlay of the initial (0 minutes; pink), and final image (7 minutes; green) from Panel D. G) Growth evolution of an N1ICD condensate using SRRF imaging, clearly demonstrating the progressive formation of a hollow core over time (180 minutes). H) Boxplot of Endogenous and OptoNotch Notch1 condensate signal widths with average widths of 194nm (+/-112) and 198nm (+/-66) respectively. N=100 condensates per condition. Scale bar 500nm. See Source Data Extended Figure 8

Extended Data Figure 9: Non-Transfected Control cells from Figure 4 panel A:

Immunofluorescence images of HEK293 treated with GSI staining for either RBPJ, MAML1, p300, MED1, BRD4, RNAPOLII, or BRD-UTP. Scale Bar 10 µm.

Extended Data Figure 10: RNA Polymerase 2 colocalizes with OptoNotch Condensates In Living Cells.

A) HEK293 cells were transfected with either DENDRA-RNAPolII (top panel), activated OptoNotch (middle), or both activated OptoNotch and DENDRA-RNAPolII (bottom) and imaged prior to UV photoconversion. B) Cells from Panel A post UV photoconversion with both 488 and 568 nm light, showing strong colocalization between green and red signals in intranuclear N1ICD condensates. C) Quantification of the ratio of red to green fluorescence in each

condition prior to UV photoconversion N=60 cells per condition. D) Quantification of the ratio of red to green fluorescence in each condition following UV photoconversion N=60 cells each per condition. E) Live fluorescence image of a GSI treated, OptoNotch (green) activated, HEK293 cell stained with live RNA dye (purple). Scale bar 1 µm. A/C Scale bar 10µm, E Scale bar 2 µm. *p<0.01 One-way ANOVA with Tukey post hoc. See Source Data Extended Figure 10.

Extended Data Figure 11: OptoNotch activates Notch target genes Hes5 And Hey1

A) Quantification of Hes5 expression by qPCR in wildtype, OptoNotch activated, GSI-treated, or OptoNotch activated and GSI-treated cells, showing a relative expression of 1(+/-0.440),14.913(+/-4.265), 0.0947(+/-0.0765), and 7.673(+/-0.639) for Hes5 respectively. B) Quantification of Hey1 expression by qPCR in wildtype, OptoNotch activated, GSI-treated, or OptoNotch activated and GSI-treated cells, showing a relative expression of 1(+/-0.651), 10.139(+/-2.887),0.00341(+/-0.0176), and 7.205(+/-1.173) for Hey1, respectively. Bar Graph represents mean +/- SD. All data acquired in HEK293 cells. *p<0.01 One-way ANOVA with Tukey post hoc. See Source Data Extended Figure 11

Extended Data Figure 12: Replicates of SRRF Co-Localization from Figure 4 I.

A) RBPJ containing condensates. B) MAML1 containing condensates C) p300 containing condensates D) BRD4 containing condensates E) RNA PolII containing condensates F) MED1 containing condensates G) BRD-UTP containing condensates. Scale Bar 500nm.

Extended Data Figure 13: Validation of Stably transfected Hes1-Live-RNA MS2.

A) HEK293 cells stably transfected with the Hes1-Live-RNA reporter, treated with either vehicle (DMSO), GSI, or GSI in conjunction with OptoNotch activation. Scale bar 5 µm. B) Quantification of the number of Hes1-Live-RNA foci per cell in cells treated with either vehicle control, GSI, or, GSI in conjunction with OptoNotch activation, showing 17.4(+/-8.1), 5.1(+/-3.6), and 20.1(+/-12) Hes1-Live-RNA foci per cell, respectively. N=300 cells per condition. C) Example of endogenously activated Stably transfected Hes1-Live-RNA cells. Scale bar 5 µm. See Source Data Extended Figure 13.

Extended Data Figure 14: ΔANK-N1ICD condensates similarly form hollow condensates.

A) Matched confocal and SRRF microscopy of ΔANK-N1ICD transfected HEK293 cell. Scale bar 10µm B) Insets of three ΔANK-N1ICD condensates showing hollow core formation under SRRF microscopy. Scale bar 500nm.

Extended Data Figure 15: N1ICD condensates contain RBPJ and MAML1: two core components of the Notch transcriptional activation complex.

A) Fluorescence immunohistochemical staining of HEK293 cells expressing ΔANK-N1ICD, showing prominent condensates co-stained for either RBPJ (top) or MAML1(bottom). Scale bar 10µm B) Zoom insets from Panel A showing individual condensates. Scale bar 2µm C) Quantification of the proportional number of ΔANK-N1ICD condensates that contain any amount of either RBPJ 0.816(+/-0.156), or MAML1 0.933(+/-0.0523). N= 250 cells per condition. $ p<0.01 on Student T-test. See Source Data Extended Figure 15

Extended Data Figure 16: MYC RNA expression in HEK293 cells.

A) RNA *in* situ hybridization of MYC RNA in combination with fluorescence immunostaining against Notch1 protein in either DMSO control, GSI-treated, or OptoNotch activated cells. Scale bar 10µm.B) Comparison of the total Nuclear MYC RNA fluorescence in either DMSO, GSI or OptoNotch activated cells showing and average of 3640(+/-2878), 198(+/-329), and 4235(+/-4231) AU of MYC RNA, respectively. N = 600 cells per condition. C) Quantification of MYC expression by qPCR in HEK293 cells treated with either vehicle, GSI or GSI+OptoNotch, showing a relative expression of MYC of 1.00(+/-0.136),0.0835(+/-0.0380), and 2.889(+/-0.881), respectively for each condition. *$p<0.01$ One-way ANOVA with Tukey post hoc. See Source Data Extended Figure 16

Supplemental Table 1: Primers and oligonucleotides used.

Movie 1:OptoNotch Activation Leading To Subsequent Nuclear Localization Of N1ICD. In HEK293 Cells Imaged Over 25 Minutes. Scale Bar 10um.

Movie 2 : N1ICD Condensate Photo-bleaching Video Over 5 Minutes. Photo-bleached area Is Encircled In Yellow.

Movie 3 : Two N1ICD Condensates Undergoing Fusion Over The Time Course Of 5 Minutes. Scale bar 1 micron

Movie 4: 3D structure of Endogenous Notch1 condensates in T-ALL cell. Render facing the X-Y plane while rotating around the Y axis(left), Render Facing the X-Z plane while rotating around the Z axis(centre), SRRF images used to produce the render(right). Scale in left and centre is in um, scale bar 500nm.

Movie 5: 3D structure of OptoNotch condensates in HEK293 cell. Render facing the X-Y plane while rotating around the Y axis(left), Render Facing the X-Z plane while rotating around the Z axis(centre), SRRF images used to produce the render(right). Scale in left and centre is in um, scale bar 1 um.

Movie 6 : N1ICD Condensate Undergoing Growth Phase Showing An Increase In The Total Volume Of A Single Condensate Over 16 Minutes. Volume On Condensate Indicated In Centre Of Condensate Measured For Each Frame. Scale bar 1 micron

Movie 7 : ΔTAD-N1ICD Condensate photo-bleaching Video Over 5 Minutes. Bleach Area Is Encircled In Yellow.

Movie 8 : ΔAnkyrin-N1ICD Condensate photo-bleaching Video Over 5 Minutes. Bleach Area Is Encircled In Yellow.
