# Supplementary material for: Notch1 Phase Separation Coupled Percolation facilitates target gene expression and enhancer looping
Source: Sci Rep. 2024 Sep 19;14:21912. doi: 10.1038/s41598-024-71634-6 (PMC11413390; doi:10.1038/s41598-024-71634-6)
Supplement: Supplementary file 44 — Supplementary Information 36. [file 41598_2024_71634_MOESM44_ESM.pdf]

Title

Notch1 Phase Separation Coupled Percolation facilitates target gene expression and enhancerlooping.

Authors

Gregory Foran[1], Ryan Douglas Hallam[1], Marvel Megaly[1], Anel Turgambayeva[1], Daniel Antfolk[2],Yifeng Li[3], Vincent C. Luca[2], Aleksandar Necakov[1,*]

[1] Department of Biological Sciences, Brock University, 1812 Sir Isaac Brock Way, St. Catharines, Ontario, Canada, L2S 3A1

[2] Department of Immunology, Moffitt Cancer Centre, Tampa, FL, USA

[3] Department of Computer Science, Brock University, 1812 Sir Isaac Brock Way, St. Catharines,Ontario, Canada, L2S 3A1

* Corresponding author please contact Aleksandar Necakov: anecakov@brocku.ca

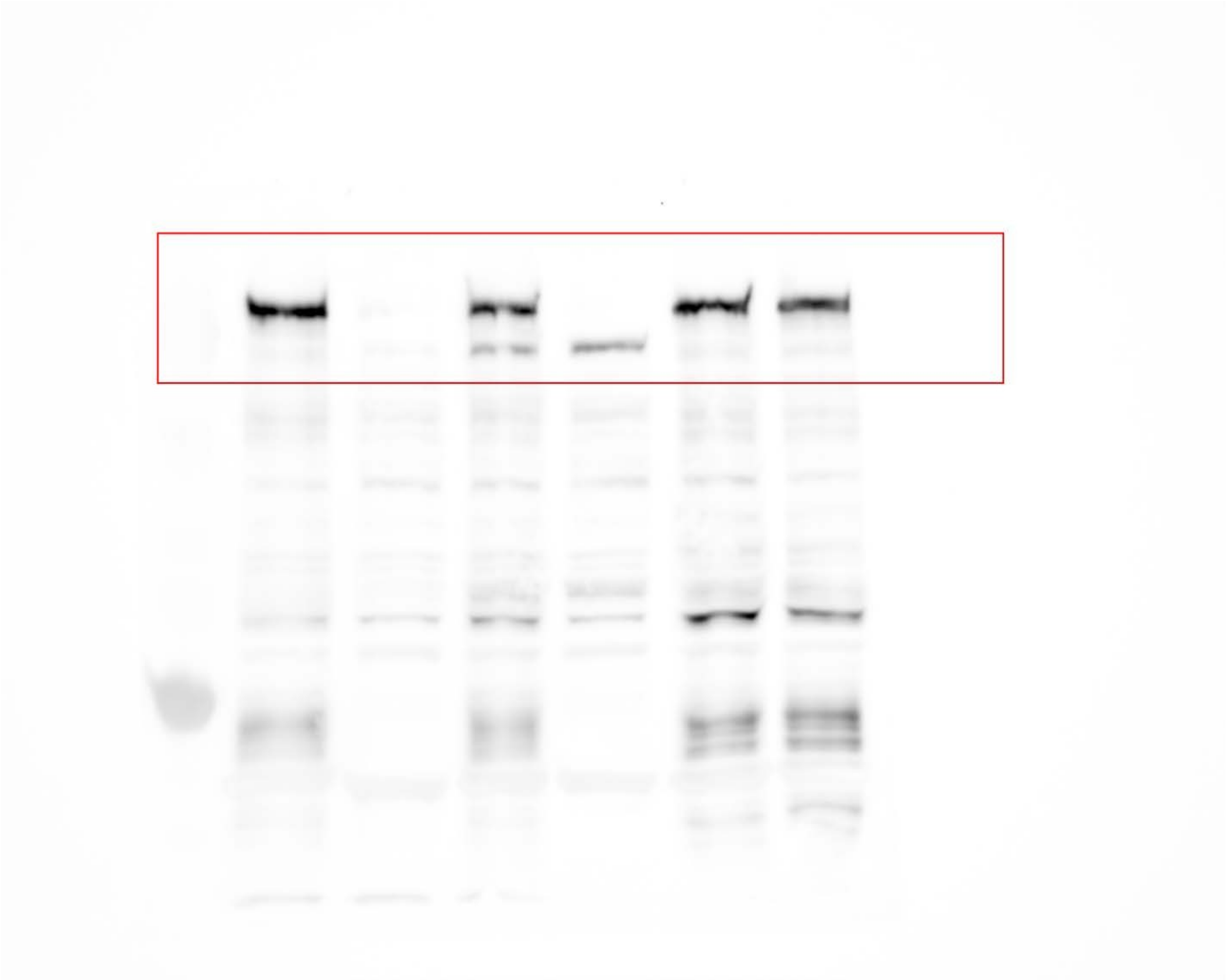

Figure 2 Western Blot of HEK293 transfected with OptoNotch with Anti-GFP antibody. Red Band represents area shown in figure with the presence of our expected bands.

A) B) C)

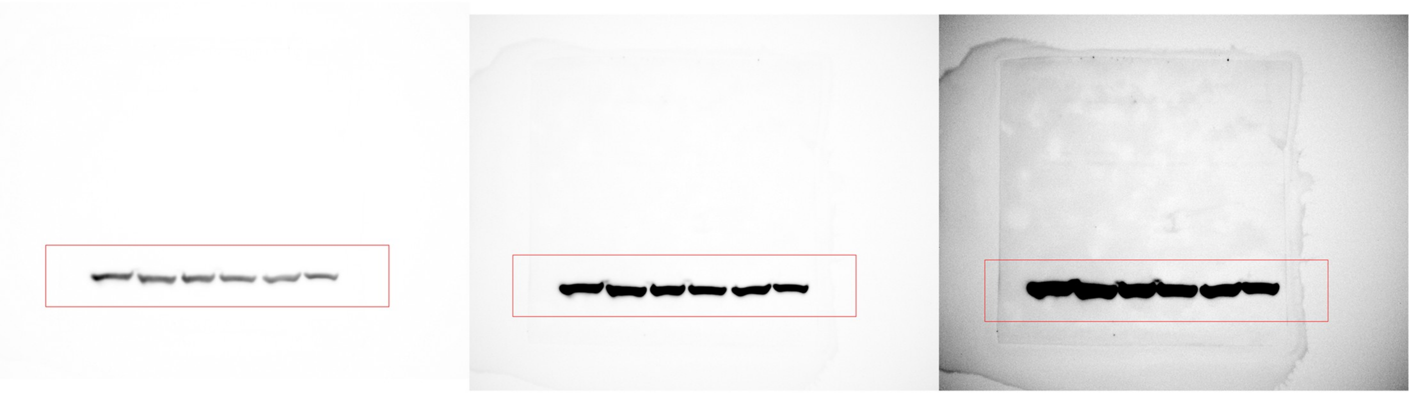

Figure 2 Western Blot of HEK293 transfected with OptoNotch with Anti-beta actin antibody. Red Band represents area shown in figure with the presence of our expected bands. A/B/C show blot at varying contrast levels.

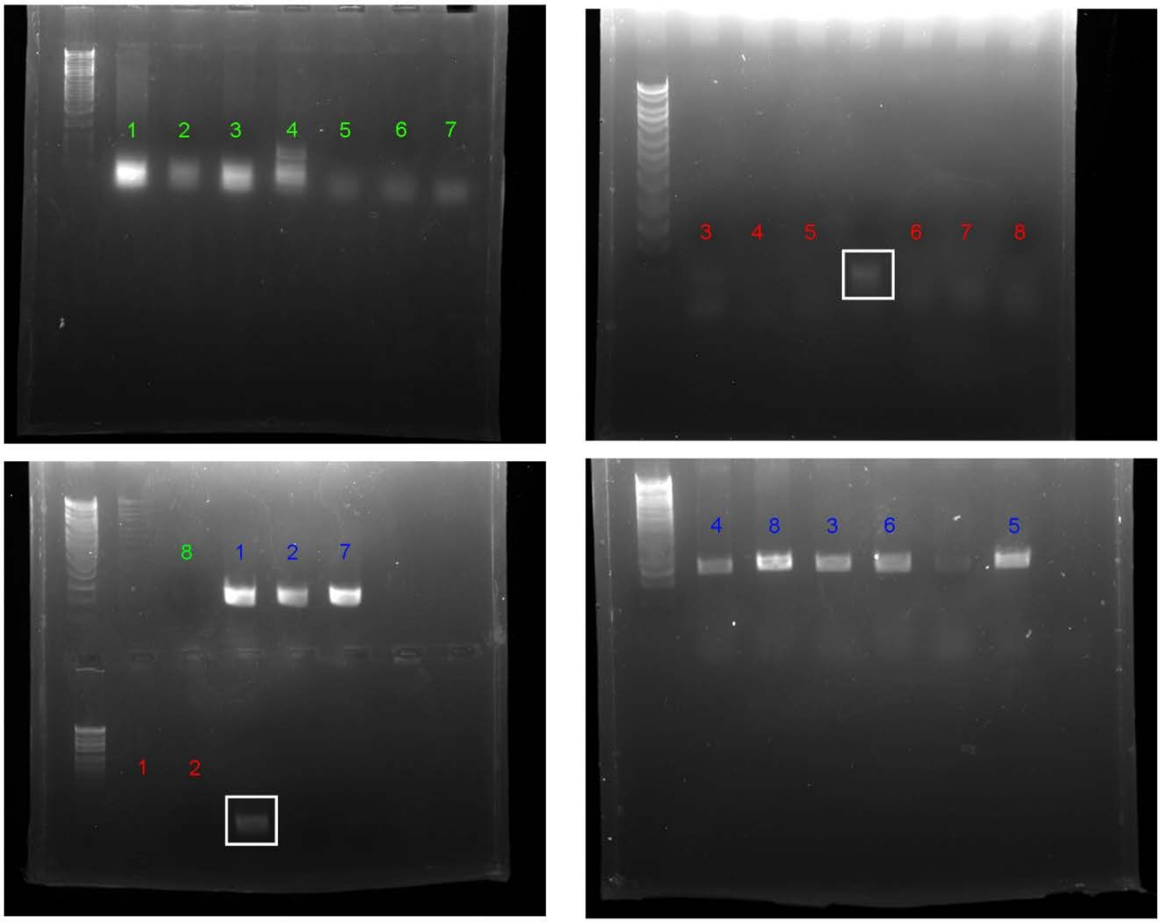

3C-PCR agarose gels used in Figure 7:

3C-PCR products for T-ALL WildType, HEK293 Wildtype, T-ALL+GSI, HEK293+GSI, T- ALL+GSI+OptoNotch, HEK293+GSI+OptoNotch, T-ALL+GSI+OptoNotch+1,6-Hexanediol, HEK293+GSI+OptoNotch+1,6-Hexanediol are represented by numbers 1-8 respectively with Green representing Myc-NDME, Blue Myc-Myc positive control, Red Myc-NDME+80K. Whiteboxes show control band presence neighboring negative controls. Lanes were re-arranged in figure for presentation.

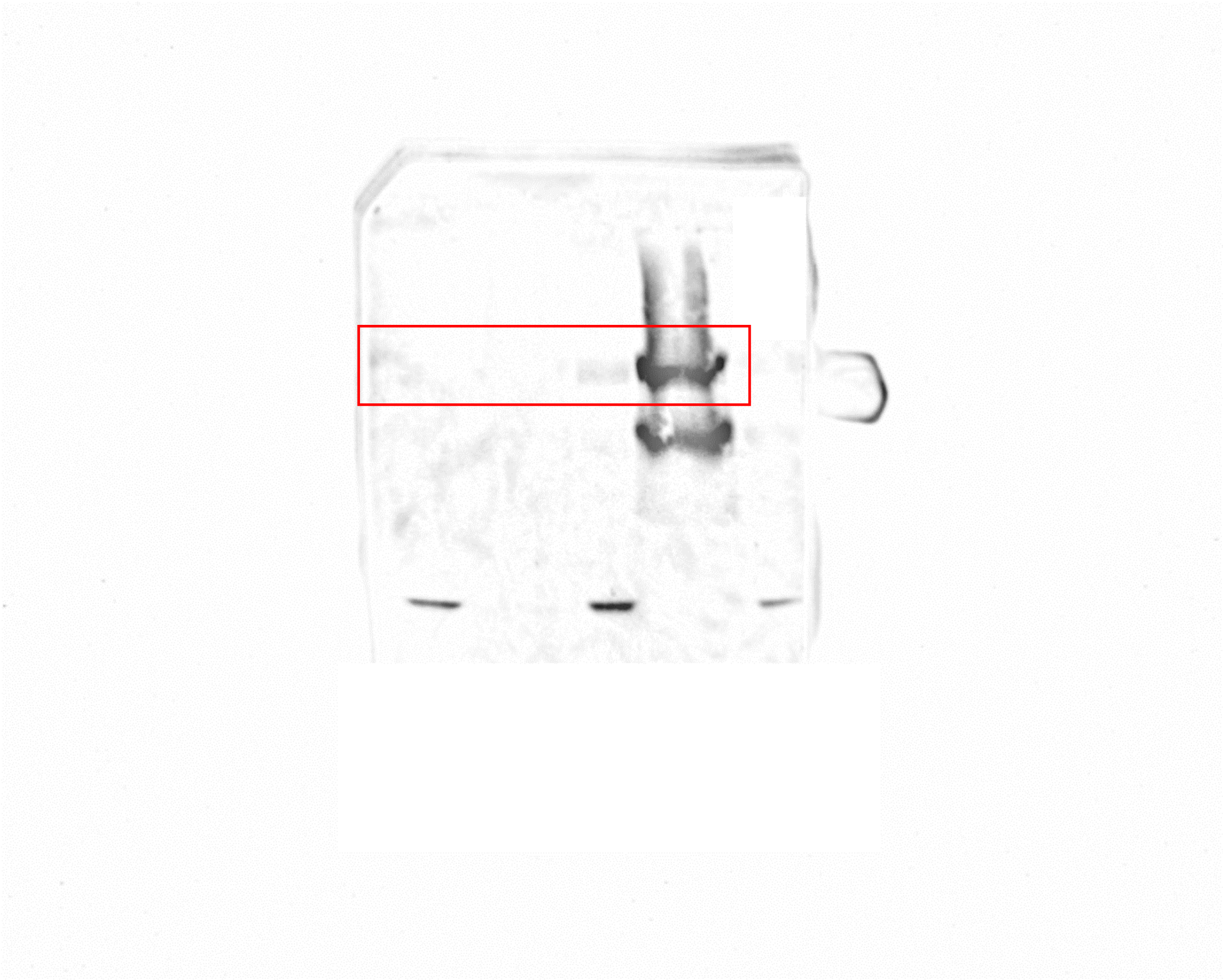

Extended Data Figure 4 Western for HEK293 for Cytoplasmic and Nuclear fractionation For endogenous Notch1 using Notch1 Antibody. Blot was inverted in figure for display purposes.

Red Box denotes area that was shown in figure.

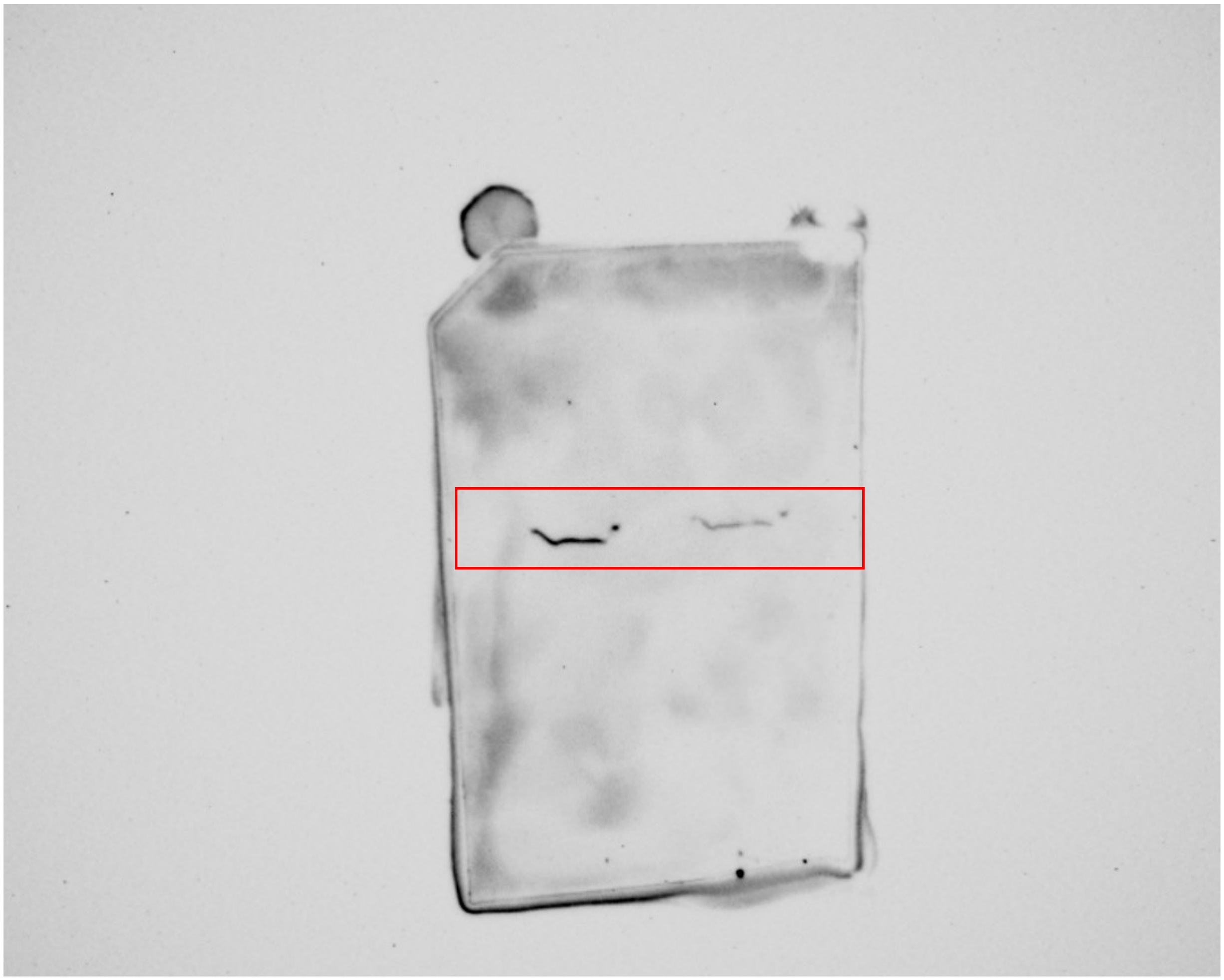

Extended Data Figure 4 Western for HEK293 for Cytoplasmic and Nuclear fractionation For endogenous Notch1 using laminin AC antibody. Blot was inverted in figure for display purposes.

Red Box denotes area that was shown in figure.

A) B) C)

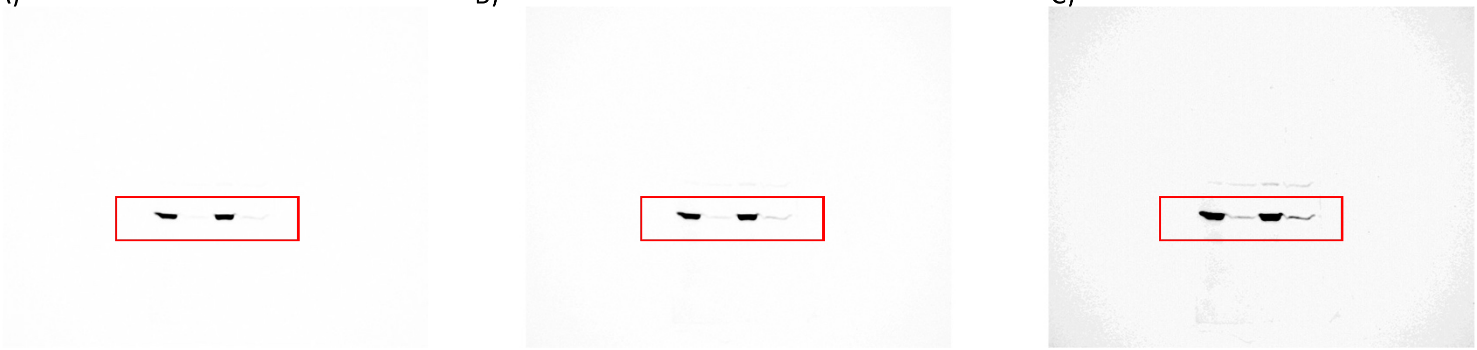

Extended Data Figure 4 Western for HEK293 for Cytoplasmic and Nuclear fractionation For endogenous Notch1 using Actin antibody. Blot was inverted in figure for display purposes. A/B/C show blot at varying contrast levels.

Red Box denotes area that was shown in figure.

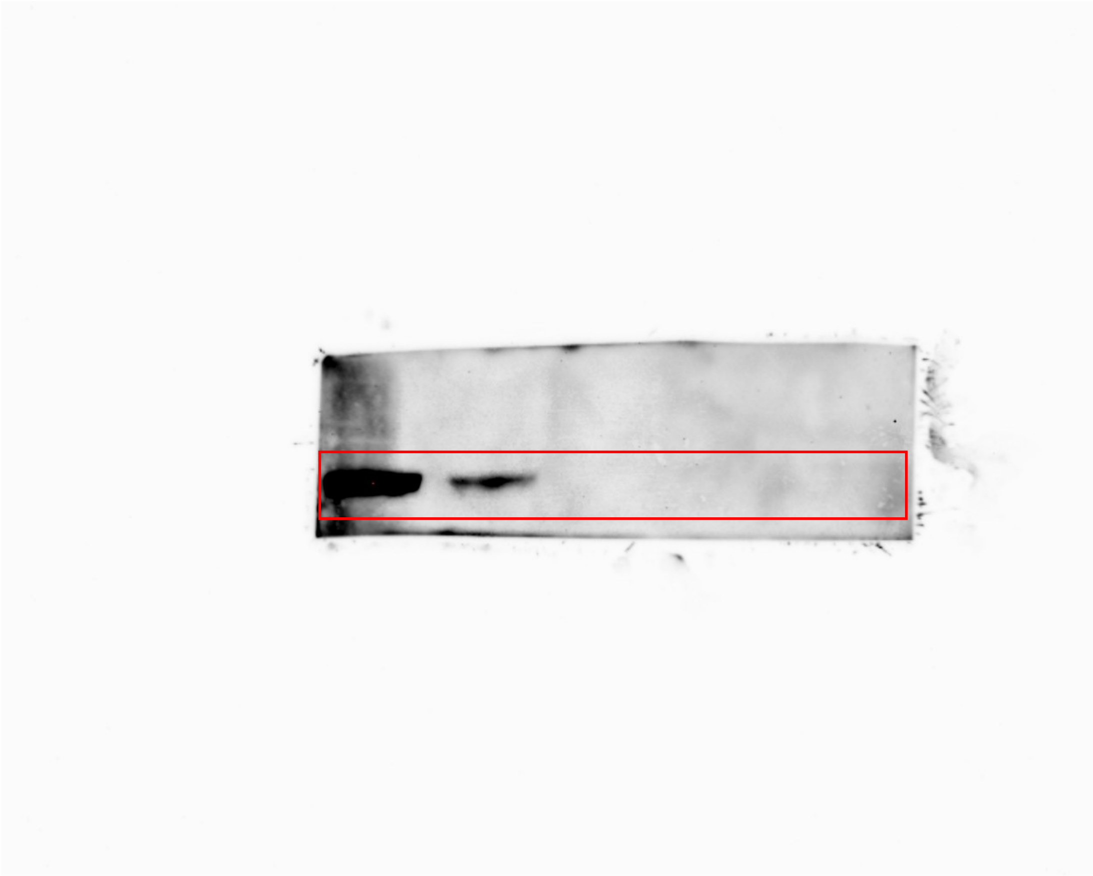

Extended Data Figure 4 Western for HEK293 cells for Cytoplasmic and Nuclear fractionation either plated on a control surface or with affixed DeltaMax for endogenous Notch1 using CST activated Notch1 antibody. Blot was inverted in figure for display purposes.

Red Box denotes area that was shown in figure.

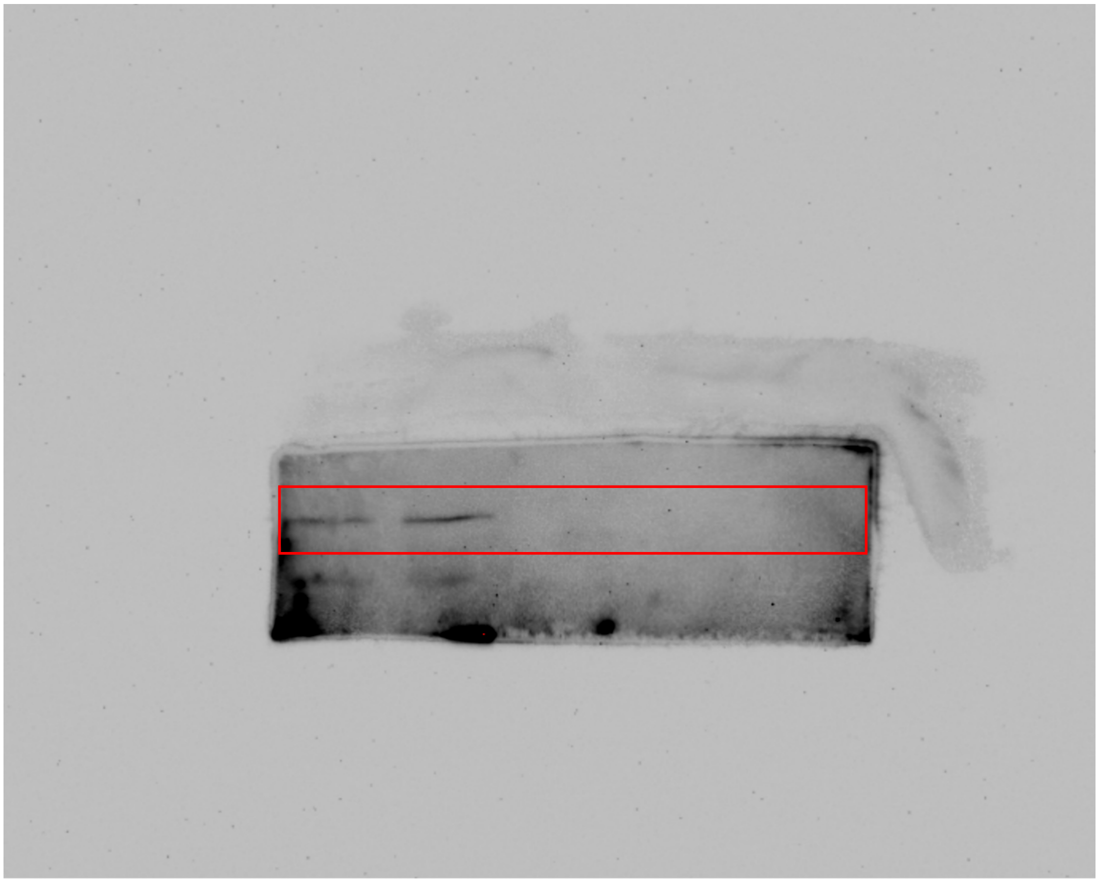

Extended Data Figure 4 Western for HEK293 cells for Cytoplasmic and Nuclear fractionation either plated on a control surface or with affixed DeltaMax for endogenous Notch1 using using laminin AC antibody. Blot was inverted in figure for display purposes.

Red Box denotes area that was shown in figure.

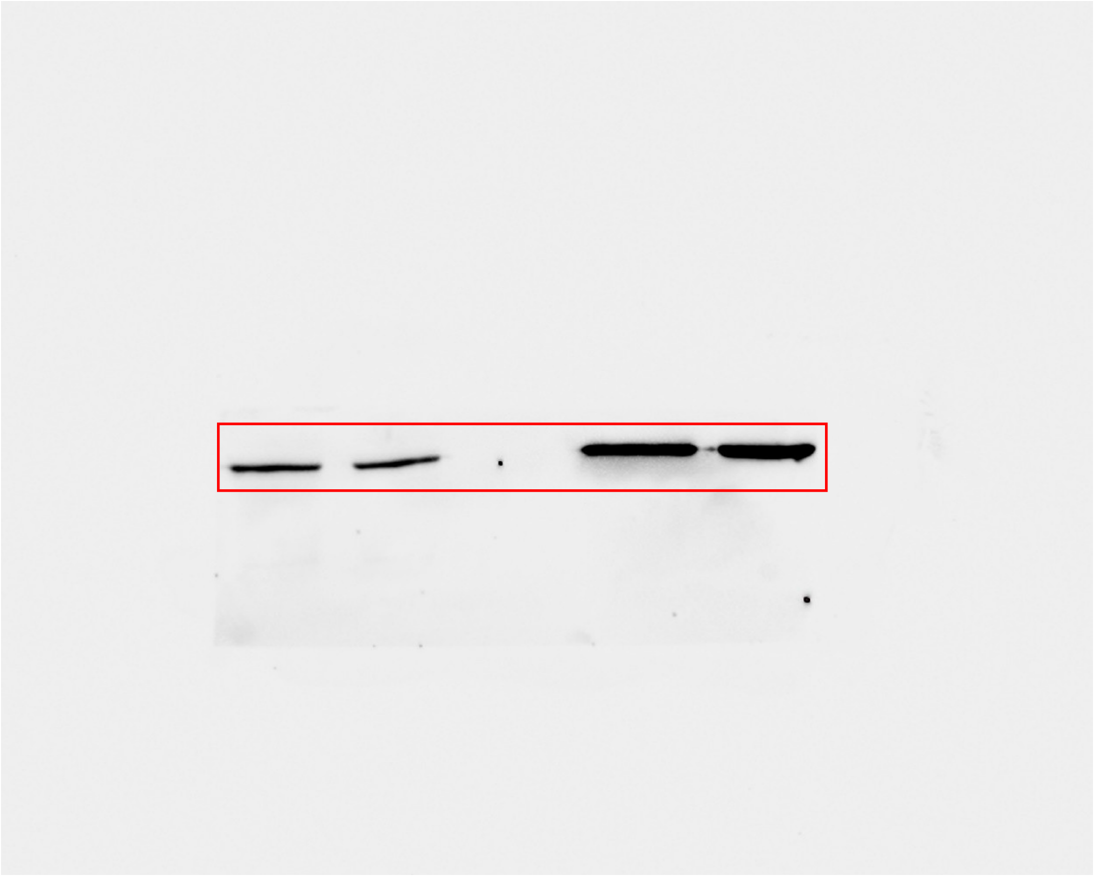

Extended Data Figure 4 Western for HEK293 cells for Cytoplasmic and Nuclear fractionation either plated on a control surface or with affixed DeltaMax for endogenous Notch1 using b-tubulin antibody. Blot was inverted in figure for display purposes.

Red Box denotes area that was shown in figure.
